# REFINING AN ICT-ENRICHED NONPHARMACOLOGICAL INTERVENTION FOR PEOPLE WITH DEMENTIA: A PARTICIPATORY DESIGN

**DOI:** 10.1093/geroni/igad104.2108

**Published:** 2023-12-21

**Authors:** Jacky C P Choy, Gloria Hoi-Yan Wong, Kayla Wong

**Affiliations:** The University of Hong Kong, Hong Kong, Hong Kong; The University of Hong Kong, Hong Kong, Hong Kong; The University of Hong Kong, Hong Kong, Hong Kong

## Abstract

Information and communication technology (ICT) has enabled the rapid development of remote non-pharmacological intervention for people living with dementia (PLwD). Yet, PLwD were seldom actively involved in the design process. We developed a prototype of Virtual Cognitive Stimulation Therapy (vCST) by adapting CST, an evidence-based group activity intervention, for remote delivery and tested among PLwD in the community. Following a participatory design approach, the current study aims to understand the needs of refinement from the users’ and providers’ perspectives for improving person-centred engagement and accessibility of vCST. Participants were 15 PLwD who received fourteen vCST sessions, 15 family carers who assisted the PLwD’s participation, and 15 trained volunteers who delivered the sessions. We observed the video-recordings of vCST sessions, and then conducted semi-structured interviews with dyads of PLwD and carers, and focus groups with the providers. Thematic analysis was conducted to understand their views on refinement needs. Two themes and 7 subthemes representing a two-level refinement needs emerged. At intervention operation level, 3 subthemes were 1) pre-assessment of personal characteristics, 2) consistent and comfortable environment and equipment setup, and 3) optimal level of carer involvement. At activity delivery level, 4 subthemes were 5) facilitating skills for positive relationships and active participation, 5) tailoring activity content to PLwD biography, interest, and ability, 6) interactive and collaborative activity design for rapport building and empowerment, and 7) materials use for enhancing stimulation and engagement. With appropriate research design and interview prompts, PLwD could comprehend novel ideas and actively contribute to intervention design.

